# Specific Detection of Two Divergent Simian Arteriviruses Using RNAscope *In Situ* Hybridization

**DOI:** 10.1371/journal.pone.0151313

**Published:** 2016-03-10

**Authors:** Shuǐqìng Yú, Yíngyún Caì, Cassandra Lyons, Reed F. Johnson, Elena Postnikova, Steven Mazur, Joshua C. Johnson, Sheli R. Radoshitzky, Adam L. Bailey, Michael Lauck, Tony L. Goldberg, David H. O’Connor, Peter B. Jahrling, Thomas C. Friedrich, Jens H. Kuhn

**Affiliations:** 1 Integrated Research Facility at Fort Detrick, National Institute of Allergy and Infectious Diseases, National Institutes of Health, Fort Detrick, Frederick, Maryland, United States of America; 2 Emerging Infectious Pathogens Section, National Institute of Allergy and Infectious Diseases, National Institutes of Health, Fort Detrick, Frederick, Maryland, United States of America; 3 United States Army Medical Research Institute of Infectious Diseases, Fort Detrick, Frederick, Maryland, United States of America; 4 University of Wisconsin-Madison, Madison, Wisconsin, United States of America; The University of Texas Medical Branch, UNITED STATES

## Abstract

Simian hemorrhagic fever (SHF) is an often lethal disease of Asian macaques. Simian hemorrhagic fever virus (SHFV) is one of at least three distinct simian arteriviruses that can cause SHF, but pathogenesis studies using modern methods have been scarce. Even seemingly straightforward studies, such as examining viral tissue and cell tropism *in vivo*, have been difficult to conduct due to the absence of standardized SHFV-specific reagents. Here we report the establishment of an *in situ* hybridization assay for the detection of SHFV and distantly related Kibale red colobus virus 1 (KRCV-1) RNA in cell culture. In addition, we detected SHFV RNA in formalin-fixed, paraffin-embedded tissues from an infected rhesus monkey (*Macaca mulatta*). The assay is easily performed and can clearly distinguish between SHFV and KRCV-1. Thus, if further developed, this assay may be useful during future studies evaluating the mechanisms by which a simian arterivirus with a restricted cell tropism can cause a lethal nonhuman primate disease similar in clinical presentation to human viral hemorrhagic fevers.

## Introduction

Simian hemorrhagic fever (SHF) is an acute viral hemorrhagic fever characterized by high lethality. Thus far, SHF has only been observed in captive Asian macaques of several species [1]. Recent genomic sequencing studies revealed that past SHF outbreaks were caused by at least three distinct simian arteriviruses (*Nidovirales*: *Arteriviridae*: *Arterivirus*). Simian hemorrhagic encephalitis virus (SHEV) caused the 1964 Sukhumi, USSR, outbreak [2], simian hemorrhagic fever virus (SHFV) caused the Bethesda, USA, epizootic outbreak only a few months later [3], and Pebjah virus (PBJV) was detected in samples collected during an SHF outbreak in Alamogordo, USA, in 1989 [2]. Several additional simian arteriviruses, among them Kibale red colobus virus 1 (KRCV-1), were recently discovered in apparently healthy African nonhuman primates [4–7]. Among them, at least KRCV-1 is able to infect and cause disease in Asian macaques in experimental settings [8].

The pathogenesis of SHF unambiguously due to SHFV (rather than PBJV, SHEV or other simian arterivirus) infection has only been evaluated in three studies during which macaques were experimentally infected with different virus isolates [9–11]. SHF is characterized by sudden onset of fever, weight loss, facial edema and erythema, dyspnea, diarrhea, lymphadenopathy, and splenomegaly. Limited intestinal and lung hemorrhages are typical manifestations, and epistaxis, hematomas, hematuria, melena, periocular hemorrhages, and petechiae are frequent findings. Disseminated intravascular coagulation, focal necroses in the liver and adrenal glands, proteinuria, lymphocyte depletion in the absence of lymphocyte infection in all lymphoid organs, and the induction of proinflammatory cytokines contribute to the severity of the disease, which ultimately progresses to shock [9–11].

Macrophages appear to be initial targets of SHFV [12]. SHFV antigen has also been detected in astrocytes, vascular endothelial cells, glial cells, and neuronal cell bodies [10]. *In vitro*, SHFV has only been shown to grow in the embryonic grivet monkey kidney MA-104 cell line and its various subclones (e.g., MARC-145, CL2621) [3, 13] and in primary macrophages and myeloid dendritic cells [12], suggesting an overall narrow cell tropism. Of the various newly discovered simian arteriviruses [4–7], only KRCV-1 was isolated in tissue culture (Wahl Jensen and Johnson *et al*., submitted). KRCV-1 growth appears to be restricted to one cell line, the MARC-145 subclone of the MA-104 cell line.

A general method for the detection of simian arteriviruses in formalin-fixed, paraffin-embedded (FFPE) tissue, independent of specific antibodies, would be a valuable tool for further characterizing the distribution of these viruses during *in vivo* infection. Conventional and tyramide-enhanced *in situ* hybridization (ISH) methods have previously been used to study the SHFV/KRCV-1-related porcine reproductive and respiratory syndrome virus (PRRSV), to evaluate virus distribution in porcine tissues following experimental exposure to PRRSV [14, 15], to differentiate PRRSV genotypes [16], and to identify coinfection of PRRSV and porcine circovirus [17]. However, no standard ISH methods have been developed for the detection of simian arterivirus RNA.

The RNAscope^®^ chromogenic assay (Advanced Cell Diagnostics, Hayward, CA) is an ISH technique that detects RNA more rapidly and with greater sensitivity than conventional ISH methods. This assay has not been previously used for the detection and quantification of RNA of any arterivirus, but has been established for several other viruses (e.g., fox circovirus [18], human immunodeficiency virus-1 [19], human papillomavirus [20], human respiratory syncytial virus [21], raccoon polyomavirus [22]). In general, RNAscope^®^ utilizes a unique probe design strategy that simultaneously amplifies signal and suppresses background to achieve single RNA molecule visualization while preserving tissue morphology [23]. A multiple set of proprietary RNAscope^®^ probe pairs (also called the “target probe”) is designed by the company on request of a client to target distinct areas of a target RNA stretch. Each probe pair consists of two oligonucleotides that readily diffuse across a variety of sample types, including cryopreserved (fresh frozen) tissue, perfused and frozen tissue, or cultured cells. Each oligonucleotide contains a region complementary and highly specific to a specific region of the RNA target, a short linker, and one half of a so-called PreAmplifier (PreAMP) sequence. The successful hybridization of both oligonucleotides of the target probe on a target RNA joins the two halves of PreAmp. So-called double z probe pairs then bind to the complete PreAMP sequence, leading to a cascade of signal amplification events that mediate the binding of label molecules that catalyze the deposition of chromogens such as diaminobenzidine (DAB). The signal detected by microscopy is thus both highly specific and highly sensitive [24]. Here, we report on the results of an RNAscope^®^ assay that can detect and differentiate between SHFV and KRCV-1, which are highly divergent simian arteriviruses [7].

## Materials and Methods

### Cells

Embryonic grivet monkey kidney MA-104 cells were obtained from the American Type Culture Collection (ATCC, Manassas, VA; #CCL-2378) and were seeded at 1 × 10^7^ cells/T175 flask and grown overnight in Eagle’s minimal essential medium (EMEM, Lonza, Walkersville, MD) supplemented with 10% heat-inactivated fetal bovine serum (FBS, Sigma-Aldrich, St. Louis, MO) at 37°C in a humidified 5% CO_2_ atmosphere. MARC-145 cells, which are derived from MA-104 cells, were obtained from Kay Faaberg (US Department of Agriculture National Animal Disease Center, Ames, IA) and grown, infected, and processed in the same way as MA-104 cells.

### Virus infection *in vitro*

We first aimed at establishment of a proof-of-principle assay, i.e., detection of SHFV *in vitro* with an SHFV-targeting RNAscope^®^ target probe. MA-104 cell growth medium was removed, and cells were exposed to EMEM or EMEM containing SHFV prototype isolate *LVR*42-0/*M*6941 [ATCC #VR-533; GenBank AF180391.2 [25, 26]], prepared as described previously [13], at a multiplicity of infection (MOI) of 0.1. Flasks were returned to the incubator and rocked every 15 min for 1 h. Inoculates were removed, and cells were washed with EMEM before adding EMEM with 10% FBS to the flasks. Cells were incubated for 24–48 h until a cytopathic effect (CPE; cell death) was observed in approximately 50–60% of cells exposed to SHFV compared to an uninfected control flask.

KRCV-1 was recently isolated in tissue culture at the Wisconsin National Primate Research Center (WNPRC) in Madison, WI [8]. KRCV-1 was amplified on MARC-145 cells prior to use as described previously [8].

### Experimental infections

Experimental infection of rhesus monkeys (*Macaca mulatta*) by intramuscular administration of SHFV *LVR*42-0/*M*6941 [GenBank KM371111 [26]] was reported previously by our laboratory [10]. We obtained >5-year-old formalin-fixed, paraffin-embedded (FFPE) brain-, liver-, and spleen-tissue sections that were derived from rhesus monkeys that had died on day 9 post-inoculation with a target dose of 5 × 10^3^ pfu of SHFV or were mock-infected.

### Slide preparation

For *in vitro* studies, supernatant was removed, 5 ml of cold phosphate-buffered saline (PBS, pH7.4, Life Technologies, Grand Island, NY) were added to each flask, and cells were harvested by scraping. Cells were transferred into labeled 50-ml conical tubes and centrifuged at 224 × *g* in a table-top centrifuge for 5 min. Cell pellets were then washed with cold PBS, resuspended in 2 ml of 10% neutral buffered formalin (Life Technologies), and fixed overnight at 4°C. Next, cells were pelleted by centrifugation, and formalin was aspirated prior to washing the cells twice with cold PBS. HistoGel (Life Technologies) was liquefied by heating at 55°C. After removal of the PBS from the sample tubes, cells were resuspended in 500 μl of HistoGel and immediately re-pelleted at 224 × *g* for 1 min in a table-top centrifuge. Supernatant-containing HistoGel was removed, and the cell pellets were solidified on ice. The resulting cell pellet-HistoGel blocks were dehydrated, embedded in paraffin following standard histology procedures, and then cut in sections of 5-μm thickness.

### *In situ* hybridization

*In situ* hybridization was performed using the RNAscope^®^ 2.0 HD Brown Chromogenic Reagent Kit according to the manufacturer’s instructions (Advanced Cell Diagnostics, Hayward, CA). Target probes with proprietary sequences were designed using custom software as described previously [23] to target the SHFV and KRCV-1 nucleocapsid (N) genes. GenBank accession numbers, target regions, and catalog order numbers for the proprietary target probes are: SHFV (GenBank AF180391.2; nucleotides 15,315–15,594; Advanced Cell Diagnostics #402481) and KRCV-1 (GenBank HQ845737; nucleotides 15,042–15,401; Advanced Cell Diagnostics #406651). Briefly, prepared slides were baked for 1 h at 60°C prior to use. After deparaffinization and hydration, tissues and cells were air-dried and treated with a peroxidase blocker before heating in a target retrieval solution (pretreatment 2 solution as part of the RNAscope^®^ kit; Advanced Cell Diagnostics #320043) for 20 min at 95–100°C. Protease (pretreatment 3 solution of the RNAscope^®^ kit; Advanced Cell Diagnostics #320045) was then applied for 30 min at 40°C. Target probes were hybridized for 2 h at 40°C, followed by a series of signal amplification and washing steps. Hybridization signals were detected by chromogenic reactions using DAB chromogen followed by 1:1 (vol/vol)-diluted hematoxylin (Fisher Scientific, Pittsburg, PA) counterstaining. Only *in vitro* samples with an average of at least 1 positive (brown) dot per cell were included for analysis. Slides were examined by microscopy (Leica Microsystems, Buffalo Grove, IL). At least 4 fields (200X-magnified images) were captured for each section using Leica Application Suite (LAS) v3.8 (Leica Microsystems). For *in situ* studies, formalin-fixed, paraffin-embedded (FFPE) tissues from SHFV-infected or mock-infected rhesus monkeys were processed as the *in vitro* slides, starting from the baking step. For statistical analysis of *in situ* samples, positive foci (most likely resembling single positive cells) were counted manually for each field, and standard deviation was calculated using GraphPad Prism 6 software (La Jolla, CA, USA).

## Results and Discussion

**Fig 1:**
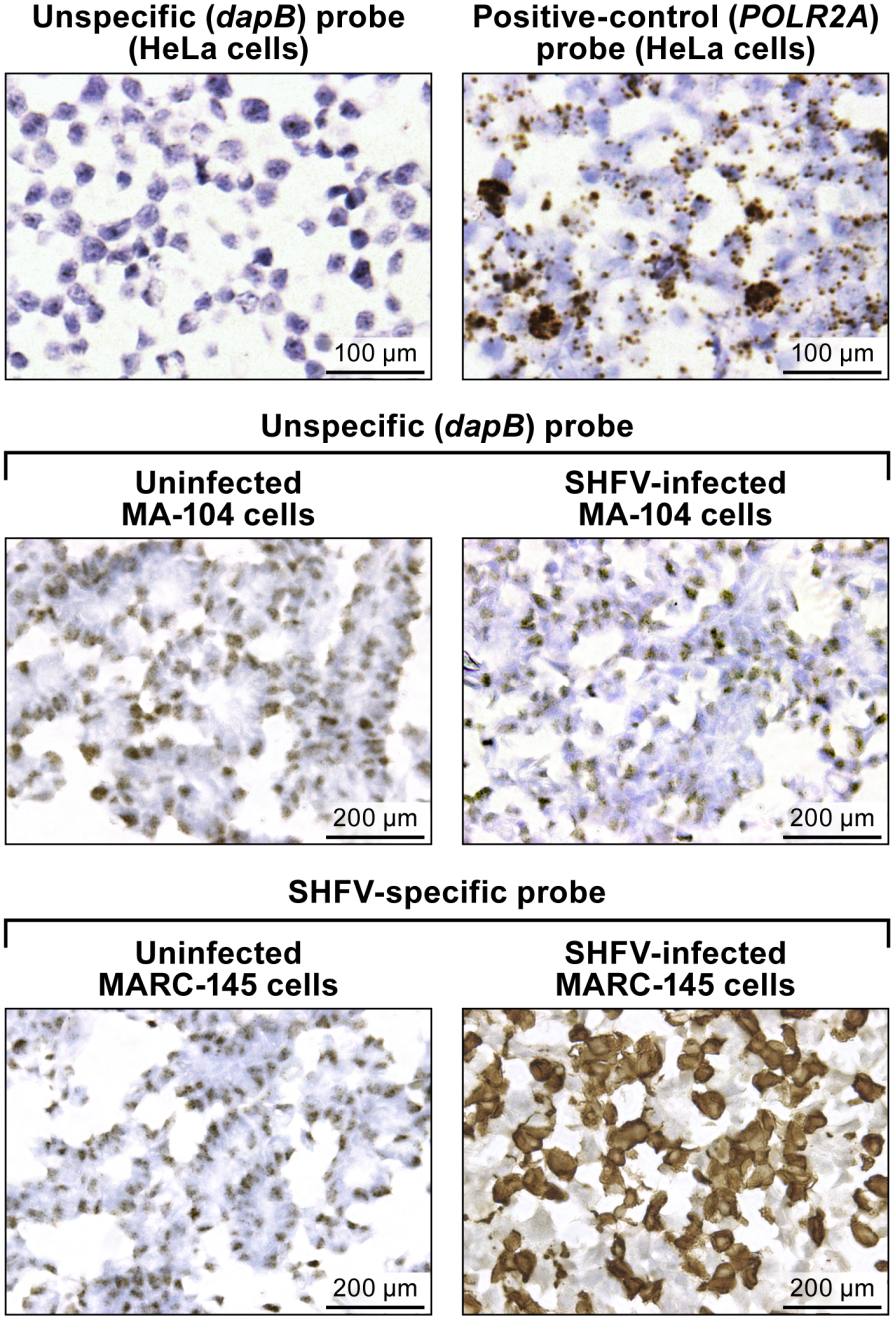
*In vitro* detection of SHFV RNA in infected cells using RNAscope^®^ *in situ* hybridization. (top): uninfected control HeLa cell slides. (top left) Result RNAscope^®^ in situ hybridization with a negative-control target probe targeting the bacterial *dapB* gene. (top right) Result with a positive-control target probe targeting the human *POLR2A* gene. (center and bottom) Uninfected- or SHFV-infected MA-104 cells treated with unspecific (*dapB*) or SHFV-specific target probes. Positive results manifest as brown staining after amplification (top right, bottom left). All images were originally taken at 400X magnification.

Staining of a control HeLa cell slide, provided by the company, with the RNAScope^®^ bacterial dihydrodipicolinate reductase (*dapB*; Advanced Cell Diagnostics #310043) negative control target probe resulted in light background staining, probably due to unspecific binding without amplification to cellular nucleic acids (Fig 1, top left). Amplification was clearly visible as brown staining on the control Hela cells slide stained with the RNAscope^®^ DNA-directed RNA polymerase II subunit RPB1 (*POLR2A*) (Advanced Cell Diagnostics #310451) positive control target probe (Fig 1, top right). Unspecific staining was also observed with the unspecific (*dapB*) control target probe on uninfected and SHFV-infected MA-104 cells (Fig 1, center) and on uninfected MARC-145 cells. Positive (brown) DAB staining was detected only in SHFV-infected MA-104 cells with the SHFV-target probe (Fig 1, bottom right).

**Fig 2:**
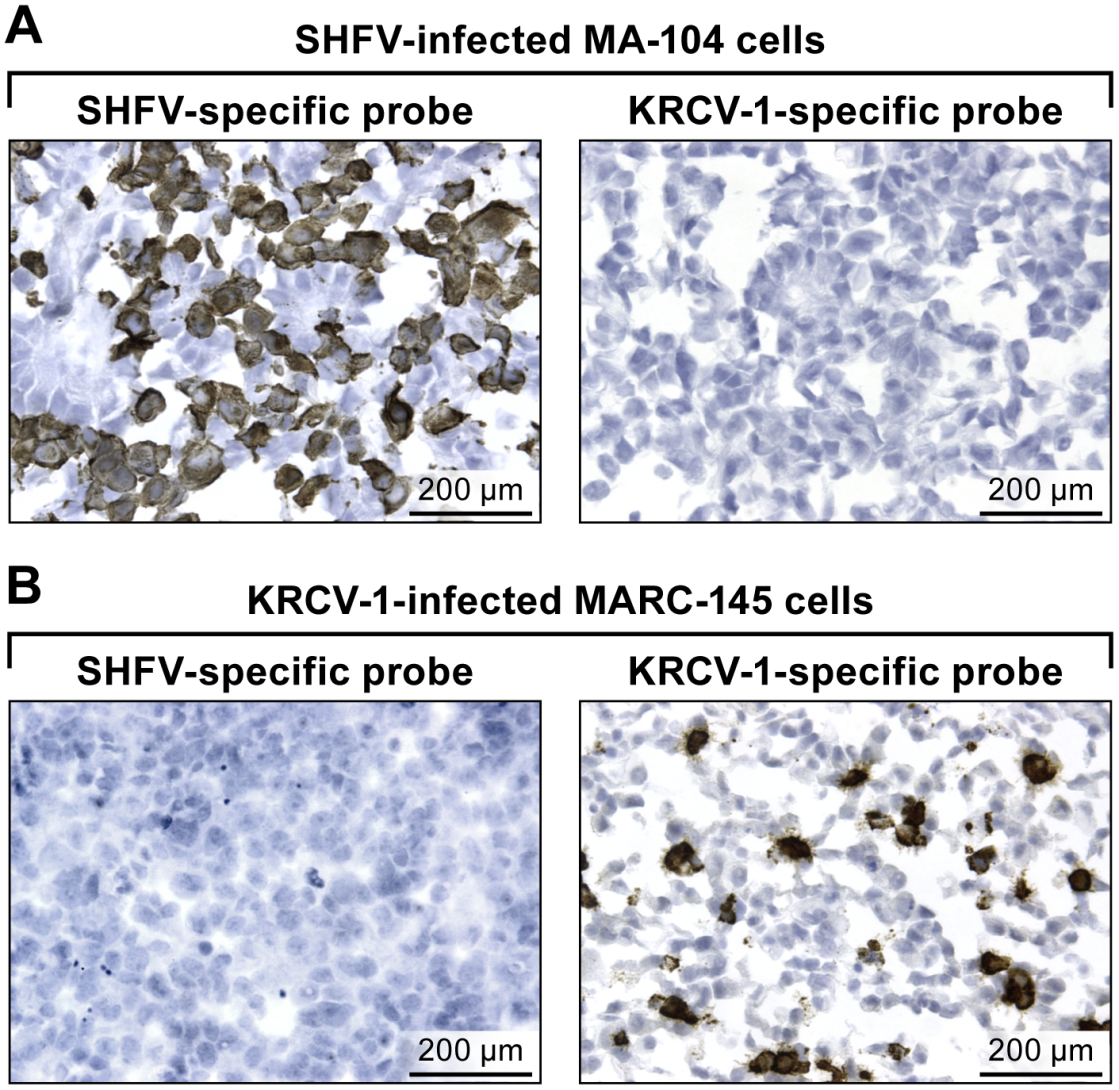
*In vitro* detection of KRCV-1 RNA in infected cells using RNAscope^®^ *in situ* hybridization. (A) SHFV-infected MA-104 cells labeled with SHFV- (left) or KRCV-1-specific (right) probes. (B) KRCV-1-infected MARC-145 cells labeled with SHFV- (left) or KRCV-1-specific (right) probes. Positive results manifest as brown staining after amplification. All images were originally taken at 400X magnification.

Using either the SHFV or the KRCV-1 target probe on SHFV-infected MA-104 cells, we show that the KRCV-1 target probe does not react with SHFV RNA (Fig 2A). On the other hand, by using MARC-145 cells infected with KRCV-1, we show that the KRCV-1 target probe detects KRCV-1 RNA, whereas the SHFV probe does not detect KRCV-1 RNA In contrast to the experiment shown in Fig 1, very little background staining was observed with the KRCV-1-specific target probe on MA-104 cells or the SHFV-specific target probe on MARC-145 cells (Fig 2B).

**Fig 3:**
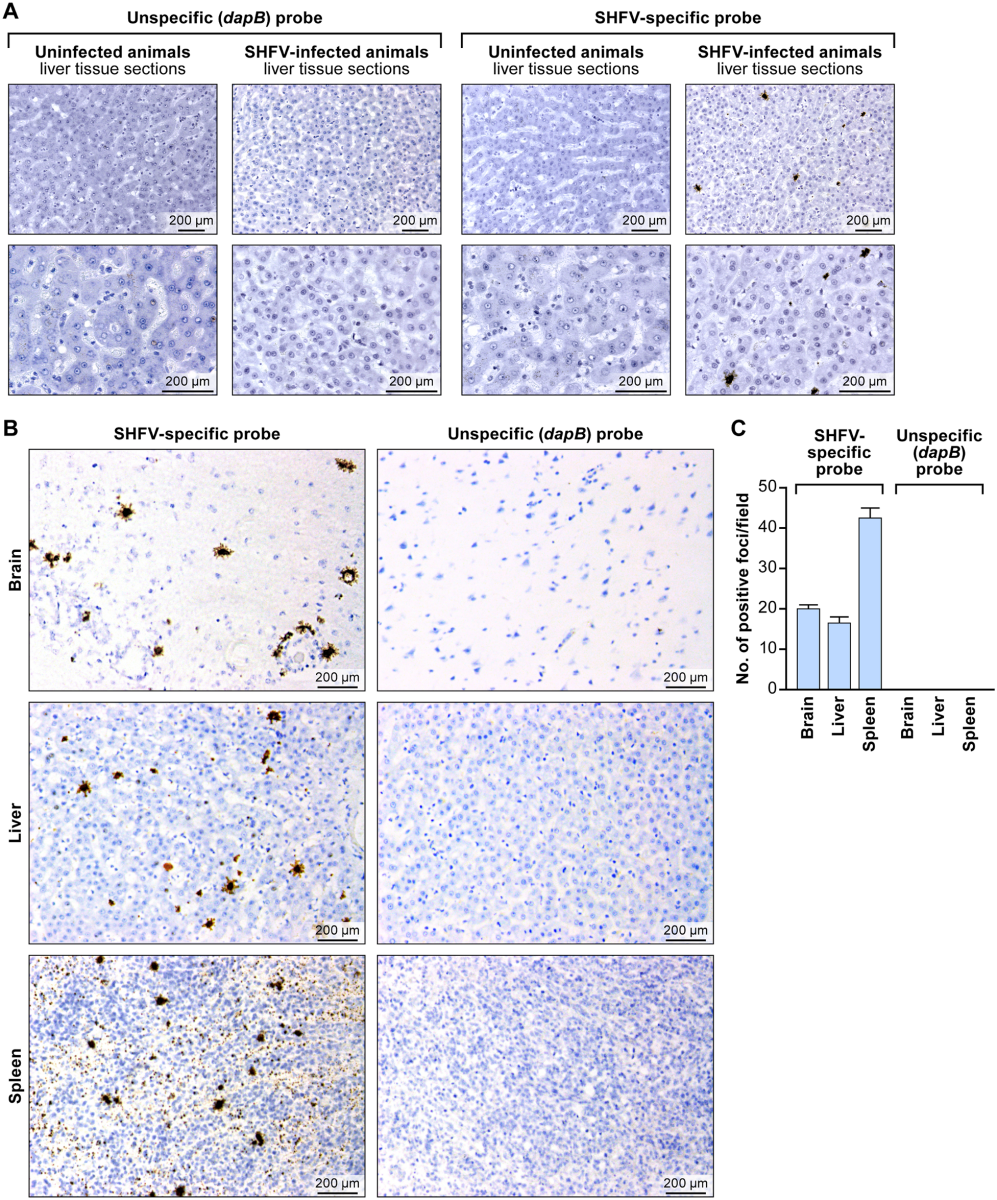
*In situ* detection of SHFV RNA from tissue sections from an SHFV-infected rhesus monkey using RNAscope^®^ *in situ* hybridization. (A) Liver sections from an uninfected or SHFV-infected rhesus monkey labeled with unspecific (*dapB*) or SHFV-specific target probes. Top: all images were originally taken at 200X magnification. Bottom: all images were originally taken at 400X magnification. Positive results manifest as brown staining. (B) Detection of SHFV RNA in brain, spleen, and additional liver sections of the same animal (original magnification 400X). Positive results manifest as brown staining after amplification. (C) Quantification of SHFV RNA-positive foci in brain, liver, and spleen sections by counting; four fields were counted per tissue section of 200X-magnified images (*p* value calculated by multiple t-test analysis with GraphPad Prism 6 software).

Next, we evaluated the usefulness of the RNAscope^®^ assay for detection of SHFV in FFPE tissues. We hybridized rhesus monkey liver sections with the SHFV target probe or control target probes as described above. Again we only detected SHFV-positive (brown) foci in samples from SHFV-infected animals exposed to the SHFV target probe. Negative results were obtained for SHFV-infected samples exposed to control target probe and for uninfected samples exposed to either target probe (Fig 3A and 3B). These results indicate that RNAscope^®^ assay is sufficiently specific to detect SHFV RNA in FFPE cells despite SHFV’s limited tissue tropism and spread.

Finally, we applied RNAscope^®^ to brain and spleen tissue sections stemming from the same SHFV-infected animal [10]. Consistent with electron-microscopic and immunohistochemical results described previously for SHFV-infected rhesus monkeys [10], positive staining was obtained for all tested tissue types (liver, spleen, brain) with the SHFV target probe, but no signal was present when the control target probe was used (Fig 3B). We quantified the number of SHFV RNA-positive foci manually using LAS v3.8 in four fields per section (Fig 3C). Our data indicate that the number of SHFV-infected cells may be higher in spleen than in brain or liver (multiple t-test analysis: *p* <0.05 for spleen compared to brain or liver).

## Conclusion

Together, our data indicate that RNAScope^®^ is a suitable assay for simian arterivirus RNA detection both *in vitro* and *in situ*, and that the assay can differentiate between SHFV and KRCV-1, which are highly divergent members of the same viral clade. Future refinements will be necessary to pinpoint and eliminate target probe sequence components that lead to unspecific background staining, and to determine the assay’s limit of detection of simian arterivirus RNAs and its specificity in context of infection with simian arteriviruses that are more closely related to each other than SHFV and KRCV-1. A similar assay developed to cover additional simian arteriviruses would be especially useful because wild red colobus monkeys have been found to be co-infected with at least two simian arteriviruses: KRCV-1 and KRCV-2 [6]. Similarly, two other simian arteriviruses, Kibale red-tailed guenon viruses 1 and 2 (KRTGV-1/2) co-circulate in a wild red-tailed guenon (*Cercopithecus ascanius*) population [7]. However, the development of such an advanced RNAscope^®^ assay will have to wait until tissue samples from infected wild primates become available or until the various simian arteriviruses other than SHFV and KRCV-1 have been isolated in cell culture and experimental animal studies have been performed. Likewise, it will be interesting to see for how long simian arterivirus RNA can be detected with RNAscope^®^ in FFPE tissues. Our study suggests that at least in the case of SHFV, detection in such tissues is possible more than 5 years post-fixation.

In the meantime, the RNAscope^®^ assay presented here could be expanded to include simultaneous detection of viral and cellular nucleic acids using a dual-color system as described in [27, 28]. Such a mixed detection assay could aid in the identification of the specific cell types infected by simian arteriviruses *in vivo/in situ* and thereby lead to a better definition of SHFV cell and tissue tropism.
